# 5′-([1,1′-Biphen­yl]-4-yl)-1′,1′′,3′′-tri­methyl­dispiro[indane-2,2′-pyrrolidine-4′,5′′-[1,3]diazin­ane]-1,3,2′′,4′′,6′′-penta­one

**DOI:** 10.1107/S1600536814013117

**Published:** 2014-06-11

**Authors:** Subramanyahegde, Hosamani Amar, Yellappa Shivaraj, Giriyapura R. Vijayakumar, Bandrehalli Siddagangaiah Palakshamurthy

**Affiliations:** a Department of Chemistry, Government Science College, Bangalore 560 001, India; b Solid State and Structural Chemistry Unit, Indian Institute of Science, Bangalore 560 012, India; c Department of Chemistry, U.C.S., Tumkur University, Tumkur, Karnataka 572 103, India; d Department of Studies and Research in Physics, U.C.S., Tumkur University, Tumkur, Karnataka 572 103, India

## Abstract

In the title compound, C_30_H_25_N_3_O_5_, the central five-membered heterocyclic ring adopts an envelope conformation, with the N atom as the flap. The dihedral angles between this central ring and the pendant indane ring system, the trione and benzene rings are 87.49 (5), 82.95 (10) and 72.42 (10)°, respectively. The dihedral angle between the rings of the biphenyl group is 45.99 (13)°. In the crystal, mol­ecules are linked by C—H⋯O hydrogen bonds into [101] *C*(12) chains.

## Related literature   

For background to multi-component or tandem reactions, see: Bunce *et al.* (2007 ▶); Duan *et al.* (2005 ▶); Ohno *et al.* (2007 ▶); Pache *et al.* (2003 ▶).
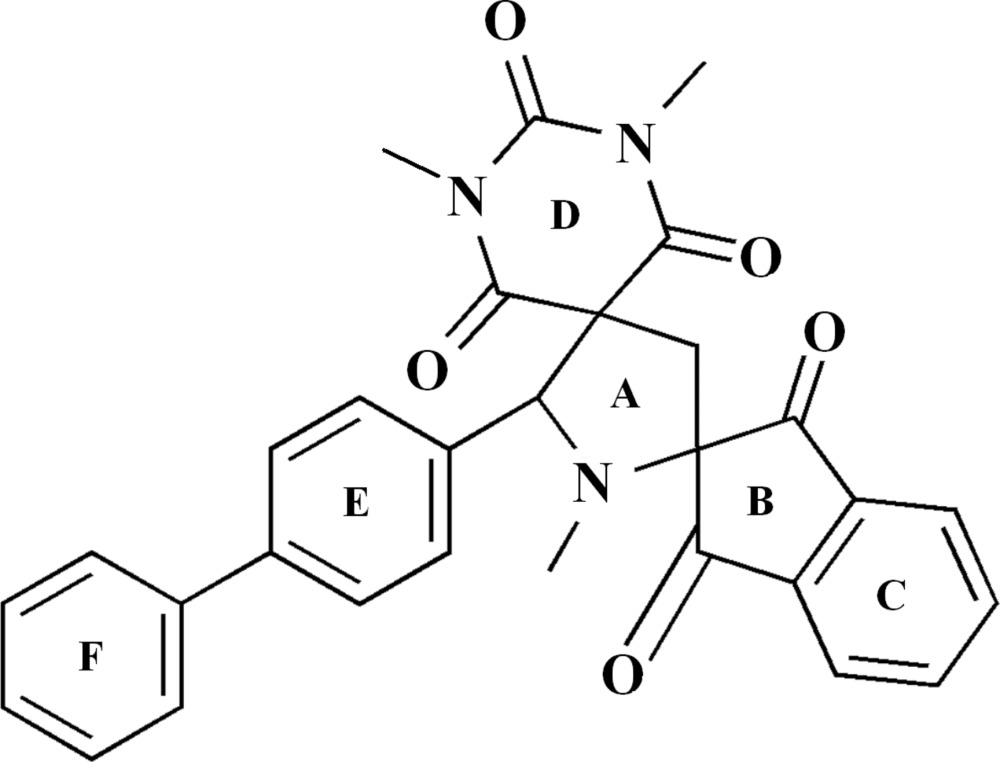


## Experimental   

### 

#### Crystal data  

C_30_H_25_N_3_O_5_
*M* *_r_* = 507.53
Monoclinic, 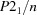

*a* = 8.3301 (10) Å
*b* = 26.070 (4) Å
*c* = 12.0441 (14) Å
β = 94.496 (6)°
*V* = 2607.5 (6) Å^3^
*Z* = 4
Mo *K*α radiation
μ = 0.09 mm^−1^
*T* = 296 K
0.24 × 0.22 × 0.20 mm

#### Data collection  

Bruker APEXII CCD diffractometer
Absorption correction: multi-scan (*SADABS*; Bruker, 2009 ▶) *T*_min_ = 0.979, *T*_max_ = 0.982
18992 measured reflections
4569 independent reflections
3332 reflections with *I* > 2σ(*I*)
*R*_int_ = 0.046

#### Refinement  

*R*[*F* ^2^ > 2σ(*F* ^2^)] = 0.050
*wR*(*F* ^2^) = 0.130
*S* = 1.04
4569 reflections
346 parameters
H-atom parameters constrained
Δρ_max_ = 0.17 e Å^−3^
Δρ_min_ = −0.15 e Å^−3^

### 

Data collection: *APEX2* (Bruker, 2009 ▶); cell refinement: *SAINT-Plus* (Bruker, 2009 ▶); data reduction: *SAINT-Plus*; program(s) used to solve structure: *SHELXS97* (Sheldrick, 2008 ▶); program(s) used to refine structure: *SHELXL97* (Sheldrick, 2008 ▶); molecular graphics: *ORTEP-3 for Windows* (Farrugia, 2012 ▶) and *Mercury* (Macrae *et al.*, 2008 ▶); software used to prepare material for publication: *SHELXL97*.

## Supplementary Material

Crystal structure: contains datablock(s) I, New_Global_Publ_Block. DOI: 10.1107/S1600536814013117/hb7231sup1.cif

Structure factors: contains datablock(s) I. DOI: 10.1107/S1600536814013117/hb7231Isup2.hkl

CCDC reference: 1006972

Additional supporting information:  crystallographic information; 3D view; checkCIF report

## Figures and Tables

**Table 1:** Hydrogen-bond geometry (Å, °)

*D*—H⋯*A*	*D*—H	H⋯*A*	*D*⋯*A*	*D*—H⋯*A*
C29—H29⋯O2^i^	0.93	2.44	3.269 (3)	149

Symmetry code: (i) 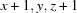.

**Fig. 1.**
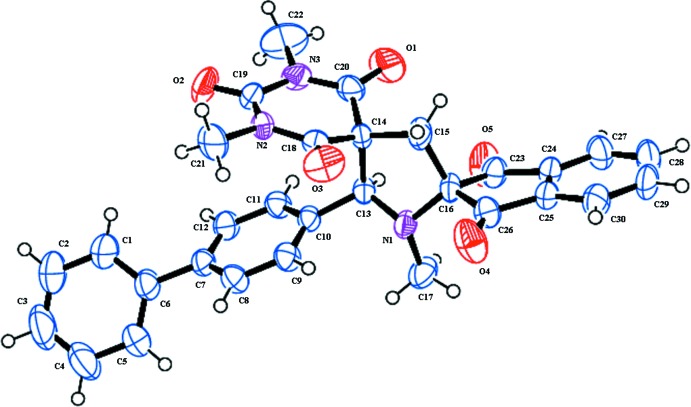
Molecular structure of the title compound, showing displacement ellipsoids drawn at the 50% probability level.

**Fig. 2.**
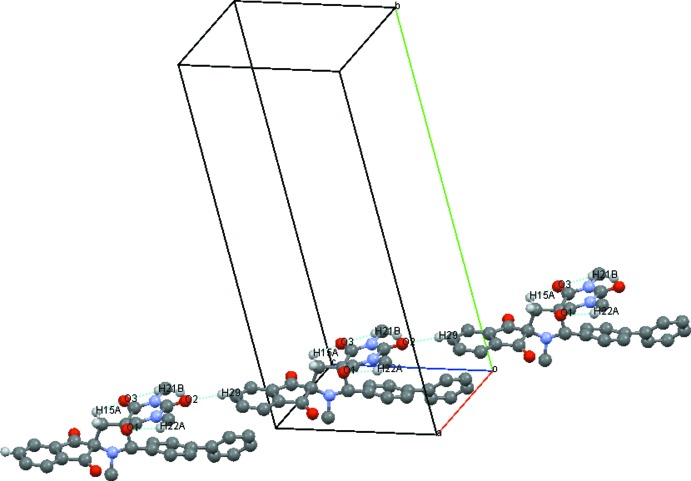
Molecular packing of the title compound. Dashed lines indicate C—H···O intramolecular and intermolecular interactions.

## supplementary crystallographic information

### S1. Comment

One strategy that potentially meets the goals of synthesis and library production is multicomponent reactions (MCRs), in which three or more starting materials are brought together to build up molecular structure and complexity (Bunce *et al.* 2007; Duan *et al.* 2005; Ohno *et al.* 2007). The tandem reactions are significant in the context of green chemistry, as they offer convenient strategy for the rapid, elegant, and convergent construction of complex organic molecules without isolating and purifying the intermediates (Pache *et al.* 2003). In this context the title compound has been synthesized by using four components such as bi phenyl carboxaldehdyde, *N*,*N*-dimethyl barbituric acid, ninhydrin and sarcosine. Also its structure has been determined.

In the title compoud, C_30_H_25_N_3_O_5_, the dihedral angles between the rings A and B,*C*,D,*E* are 88.73 (11)°, 86.80 (11)°, 82.95 (12)°, 72.42 (10)° respectively, and between A and F, E and F are 45.99 (13)° and 36.43 (13)° respectively. In the crystal, molecules are linked by C29—H29···O2 hydrogen bonds along [101] shown in Fig.2.

### S2. Experimental

To a suspension of biphenyl carboxaldehdyde, (1.0 mmol) in MeOH was added *N*,*N*-dimethyl barbituric acid (1.0 mmol), ninhydrin (1.0 mmol), sarcosine (1.0 mmol) and magnesium silicate catalyst (5 mol %) at room temperature. The reaction mass was stirred at 60°C for 20 minutes. Title compound was precipitated as yellow solid on standing the reaction mass. Then it was filtered and washed with cold methanol to remove polar impurities. The compound was further re-crystallized using dichloromethane: methanol: tetrahydrofuron (3:1:1) to yield colourless prisms.

### S3. Refinement

The H atoms were positioned with idealized geometry using a riding model with C—H = 0.93–0.96 Å. All H atoms were refined with isotropic displacement parameters (set to 1.2–1.5 times of the *U*_eq_ of the parent atom).

### Crystal data

**Table:**

C_30_H_25_N_3_O_5_	Prism
*M**_r_* = 507.53	*D*_x_ = 1.293 Mg m^−^^3^
Monoclinic, *P*2_1_/*n*	Melting point: 434 K
Hall symbol: -P 2yn	Mo *K*α radiation, λ = 0.71073 Å
*a* = 8.3301 (10) Å	Cell parameters from 346 reflections
*b* = 26.070 (4) Å	θ = 1.9–25.0°
*c* = 12.0441 (14) Å	µ = 0.09 mm^−^^1^
β = 94.496 (6)°	*T* = 296 K
*V* = 2607.5 (6) Å^3^	Prism, colourless
*Z* = 4	0.24 × 0.22 × 0.20 mm
*F*(000) = 1064	

### Data collection

**Table:**

Bruker APEXII CCD diffractometer	4569 independent reflections
Radiation source: fine-focus sealed tube	3332 reflections with *I* > 2σ(*I*)
Graphite monochromator	*R*_int_ = 0.046
Detector resolution: 1.6 pixels mm^-1^	θ_max_ = 25.0°, θ_min_ = 1.9°
ω scans	*h* = −9→9
Absorption correction: multi-scan (*SADABS*; Bruker, 2009)	*k* = −31→30
*T*_min_ = 0.979, *T*_max_ = 0.982	*l* = −14→10
18992 measured reflections	

### Refinement

**Table:**

Refinement on *F*^2^	Primary atom site location: structure-invariant direct methods
Least-squares matrix: full	Secondary atom site location: difference Fourier map
*R*[*F*^2^ > 2σ(*F*^2^)] = 0.050	Hydrogen site location: inferred from neighbouring sites
*wR*(*F*^2^) = 0.130	H-atom parameters constrained
*S* = 1.04	*w* = 1/[σ^2^(*F*_o_^2^) + (0.059*P*)^2^ + 0.5965*P*] where *P* = (*F*_o_^2^ + 2*F*_c_^2^)/3
4569 reflections	(Δ/σ)_max_ = 0.031
346 parameters	Δρ_max_ = 0.17 e Å^−^^3^
0 restraints	Δρ_min_ = −0.15 e Å^−^^3^
0 constraints	

### Special details

**Table:**

Geometry. All e.s.d.'s (except the e.s.d. in the dihedral angle between two l.s. planes) are estimated using the full covariance matrix. The cell e.s.d.'s are taken into account individually in the estimation of e.s.d.'s in distances, angles and torsion angles; correlations between e.s.d.'s in cell parameters are only used when they are defined by crystal symmetry. An approximate (isotropic) treatment of cell e.s.d.'s is used for estimating e.s.d.'s involving l.s. planes.
Refinement. Refinement of *F*^2^ against ALL reflections. The weighted *R*-factor *wR* and goodness of fit *S* are based on *F*^2^, conventional *R*-factors *R* are based on *F*, with *F* set to zero for negative *F*^2^. The threshold expression of *F*^2^ > σ(*F*^2^) is used only for calculating *R*-factors(gt) *etc*. and is not relevant to the choice of reflections for refinement. *R*-factors based on *F*^2^ are statistically about twice as large as those based on *F*, and *R*- factors based on ALL data will be even larger.

### Fractional atomic coordinates and isotropic or equivalent isotropic displacement parameters (Å^2^)

**Table:**

	*x*	*y*	*z*	*U*_iso_*/*U*_eq_	
C1	0.4231 (4)	0.04651 (12)	0.1141 (2)	0.0948 (10)	
H1	0.4813	0.0729	0.0843	0.114*	
C2	0.2878 (5)	0.02719 (16)	0.0539 (3)	0.1201 (14)	
H2	0.2547	0.0412	−0.0152	0.144*	
C3	0.2029 (4)	−0.01227 (15)	0.0952 (3)	0.1017 (11)	
H3	0.1124	−0.0252	0.0545	0.122*	
C4	0.2509 (3)	−0.03246 (11)	0.1956 (3)	0.0855 (9)	
H4	0.1941	−0.0597	0.2235	0.103*	
C5	0.3845 (3)	−0.01285 (10)	0.2575 (2)	0.0675 (7)	
H5	0.4153	−0.0268	0.3269	0.081*	
C6	0.4724 (3)	0.02708 (8)	0.21760 (18)	0.0546 (6)	
C7	0.6154 (2)	0.04800 (7)	0.28376 (16)	0.0448 (5)	
C8	0.6096 (3)	0.05802 (8)	0.39615 (17)	0.0480 (5)	
H8	0.5163	0.0505	0.4306	0.058*	
C9	0.7399 (2)	0.07903 (8)	0.45822 (16)	0.0444 (5)	
H9	0.7323	0.0862	0.5333	0.053*	
C10	0.8821 (2)	0.08962 (7)	0.40952 (15)	0.0373 (4)	
C11	0.8908 (2)	0.07776 (7)	0.29854 (16)	0.0441 (5)	
H11	0.9864	0.0831	0.2652	0.053*	
C12	0.7582 (3)	0.05790 (8)	0.23626 (16)	0.0488 (5)	
H12	0.7653	0.0511	0.1610	0.059*	
C13	1.0205 (2)	0.11690 (7)	0.47311 (15)	0.0371 (4)	
H13	1.1203	0.1076	0.4400	0.045*	
C14	1.0065 (2)	0.17788 (7)	0.47424 (15)	0.0361 (4)	
C15	1.0975 (3)	0.19325 (8)	0.58443 (18)	0.0591 (6)	
H15A	1.0292	0.2137	0.6288	0.071*	
H15B	1.1924	0.2131	0.5709	0.071*	
C16	1.1458 (2)	0.14280 (7)	0.64526 (15)	0.0388 (5)	
C17	1.0825 (3)	0.05128 (8)	0.61581 (19)	0.0608 (6)	
H17A	0.9951	0.0293	0.5898	0.091*	
H17B	1.1040	0.0471	0.6948	0.091*	
H17C	1.1769	0.0424	0.5791	0.091*	
C18	0.8320 (2)	0.19348 (7)	0.47277 (16)	0.0374 (4)	
C19	0.8226 (2)	0.19976 (7)	0.27108 (17)	0.0443 (5)	
C20	1.0853 (2)	0.19684 (8)	0.37413 (19)	0.0486 (5)	
C21	0.5729 (2)	0.20337 (10)	0.3638 (2)	0.0740 (8)	
H21A	0.5428	0.2389	0.3578	0.111*	
H21B	0.5337	0.1890	0.4299	0.111*	
H21C	0.5271	0.1851	0.2998	0.111*	
C22	1.0650 (4)	0.20798 (13)	0.1717 (2)	0.0980 (11)	
H22A	1.1795	0.2041	0.1848	0.147*	
H22B	1.0408	0.2417	0.1430	0.147*	
H22C	1.0241	0.1828	0.1186	0.147*	
C23	1.3283 (2)	0.13417 (8)	0.64229 (16)	0.0459 (5)	
C24	1.4062 (2)	0.14997 (8)	0.75118 (16)	0.0426 (5)	
C25	1.2897 (2)	0.15729 (8)	0.82564 (15)	0.0415 (5)	
C26	1.1278 (3)	0.14805 (8)	0.77001 (17)	0.0452 (5)	
C27	1.5684 (3)	0.15617 (9)	0.7833 (2)	0.0592 (6)	
H27	1.6460	0.1515	0.7328	0.071*	
C28	1.6111 (3)	0.16939 (10)	0.8916 (2)	0.0666 (7)	
H28	1.7192	0.1739	0.9151	0.080*	
C29	1.4958 (3)	0.17615 (9)	0.96614 (19)	0.0648 (7)	
H29	1.5281	0.1846	1.0395	0.078*	
C30	1.3337 (3)	0.17078 (9)	0.93507 (17)	0.0555 (6)	
H30	1.2567	0.1760	0.9858	0.067*	
N1	1.03912 (19)	0.10473 (6)	0.59094 (12)	0.0416 (4)	
N2	0.75059 (17)	0.19896 (6)	0.36977 (13)	0.0405 (4)	
N3	0.9891 (2)	0.20081 (7)	0.27705 (13)	0.0474 (4)	
O1	1.22834 (18)	0.20472 (8)	0.37604 (18)	0.0910 (7)	
O2	0.7449 (2)	0.20158 (7)	0.18241 (13)	0.0766 (5)	
O3	0.7628 (2)	0.19887 (6)	0.55632 (13)	0.0671 (5)	
O4	1.00353 (19)	0.14533 (7)	0.81522 (14)	0.0754 (5)	
O5	1.3934 (2)	0.11654 (8)	0.56487 (13)	0.0754 (5)	

### Atomic displacement parameters (Å^2^)

**Table:**

	*U*^11^	*U*^22^	*U*^33^	*U*^12^	*U*^13^	*U*^23^
C1	0.103 (2)	0.093 (2)	0.0800 (19)	−0.0288 (18)	−0.0449 (17)	0.0214 (16)
C2	0.116 (3)	0.138 (3)	0.095 (2)	−0.036 (3)	−0.065 (2)	0.022 (2)
C3	0.077 (2)	0.112 (3)	0.108 (3)	−0.0180 (19)	−0.0423 (19)	−0.019 (2)
C4	0.0650 (18)	0.080 (2)	0.108 (2)	−0.0235 (15)	−0.0125 (16)	−0.0150 (17)
C5	0.0614 (16)	0.0671 (16)	0.0712 (16)	−0.0162 (13)	−0.0133 (12)	−0.0010 (12)
C6	0.0556 (14)	0.0499 (14)	0.0552 (14)	−0.0050 (11)	−0.0140 (11)	−0.0057 (10)
C7	0.0501 (13)	0.0386 (11)	0.0436 (12)	−0.0037 (9)	−0.0100 (10)	−0.0022 (9)
C8	0.0472 (12)	0.0475 (13)	0.0489 (13)	−0.0074 (9)	0.0011 (10)	−0.0066 (9)
C9	0.0503 (13)	0.0459 (12)	0.0368 (11)	−0.0043 (9)	0.0011 (9)	−0.0090 (9)
C10	0.0414 (11)	0.0326 (10)	0.0366 (11)	0.0010 (8)	−0.0052 (8)	−0.0041 (8)
C11	0.0482 (12)	0.0438 (12)	0.0405 (11)	−0.0052 (9)	0.0035 (9)	−0.0051 (9)
C12	0.0621 (15)	0.0497 (13)	0.0334 (11)	−0.0071 (10)	−0.0047 (10)	−0.0070 (9)
C13	0.0348 (10)	0.0376 (11)	0.0377 (11)	0.0036 (8)	−0.0053 (8)	−0.0042 (8)
C14	0.0318 (10)	0.0367 (10)	0.0383 (11)	−0.0020 (8)	−0.0072 (8)	−0.0005 (8)
C15	0.0684 (15)	0.0416 (12)	0.0617 (14)	−0.0013 (11)	−0.0314 (12)	−0.0056 (10)
C16	0.0375 (11)	0.0425 (11)	0.0346 (10)	0.0028 (8)	−0.0087 (8)	−0.0050 (8)
C17	0.0794 (17)	0.0399 (13)	0.0595 (14)	0.0090 (11)	−0.0176 (12)	0.0023 (10)
C18	0.0370 (11)	0.0369 (11)	0.0392 (11)	−0.0003 (8)	0.0085 (9)	−0.0010 (8)
C19	0.0481 (12)	0.0406 (12)	0.0422 (12)	0.0012 (9)	−0.0093 (10)	0.0071 (9)
C20	0.0295 (11)	0.0468 (12)	0.0697 (15)	0.0001 (9)	0.0061 (10)	0.0076 (10)
C21	0.0256 (11)	0.0816 (19)	0.114 (2)	0.0065 (11)	−0.0020 (12)	0.0164 (15)
C22	0.118 (3)	0.111 (3)	0.072 (2)	−0.002 (2)	0.0548 (19)	0.0207 (17)
C23	0.0441 (12)	0.0581 (13)	0.0353 (11)	0.0041 (10)	0.0020 (9)	−0.0021 (9)
C24	0.0391 (11)	0.0507 (12)	0.0367 (11)	0.0021 (9)	−0.0056 (9)	0.0013 (9)
C25	0.0423 (12)	0.0463 (12)	0.0346 (11)	−0.0008 (9)	−0.0047 (9)	−0.0005 (8)
C26	0.0406 (12)	0.0497 (13)	0.0450 (12)	0.0009 (9)	0.0017 (10)	−0.0080 (9)
C27	0.0406 (12)	0.0716 (16)	0.0637 (15)	−0.0025 (11)	−0.0063 (11)	−0.0014 (12)
C28	0.0497 (14)	0.0745 (18)	0.0712 (17)	−0.0038 (12)	−0.0240 (13)	−0.0008 (13)
C29	0.0780 (18)	0.0662 (16)	0.0448 (13)	−0.0073 (13)	−0.0293 (13)	−0.0023 (11)
C30	0.0633 (15)	0.0658 (15)	0.0363 (12)	−0.0047 (12)	−0.0033 (10)	−0.0063 (10)
N1	0.0478 (10)	0.0366 (9)	0.0380 (9)	0.0034 (7)	−0.0120 (7)	−0.0025 (7)
N2	0.0240 (8)	0.0466 (10)	0.0501 (10)	0.0027 (7)	−0.0020 (7)	0.0057 (7)
N3	0.0466 (10)	0.0558 (11)	0.0416 (10)	−0.0023 (8)	0.0144 (8)	0.0086 (8)
O1	0.0269 (9)	0.1062 (15)	0.1406 (18)	−0.0070 (9)	0.0098 (10)	0.0395 (12)
O2	0.0916 (13)	0.0810 (13)	0.0514 (10)	0.0028 (10)	−0.0321 (9)	0.0140 (8)
O3	0.0786 (12)	0.0704 (11)	0.0564 (10)	0.0113 (9)	0.0313 (9)	−0.0048 (8)
O4	0.0454 (10)	0.1172 (15)	0.0652 (11)	−0.0114 (9)	0.0141 (8)	−0.0243 (10)
O5	0.0573 (10)	0.1211 (15)	0.0485 (10)	0.0129 (10)	0.0084 (8)	−0.0239 (9)

### Geometric parameters (Å, º)

**Table:**

C1—C6	1.378 (3)	C17—H17A	0.9600
C1—C2	1.386 (4)	C17—H17B	0.9600
C1—H1	0.9300	C17—H17C	0.9600
C2—C3	1.365 (5)	C18—O3	1.206 (2)
C2—H2	0.9300	C18—O3	1.206 (2)
C3—C4	1.350 (4)	C18—O3	1.206 (2)
C3—H3	0.9300	C18—N2	1.374 (2)
C4—C5	1.387 (3)	C19—O2	1.205 (2)
C4—H4	0.9300	C19—N2	1.373 (3)
C5—C6	1.381 (3)	C19—N3	1.383 (3)
C5—H5	0.9300	C20—O1	1.208 (2)
C6—C7	1.484 (3)	C20—O1	1.208 (2)
C7—C8	1.383 (3)	C20—N3	1.369 (3)
C7—C12	1.384 (3)	C21—N2	1.480 (2)
C8—C9	1.382 (3)	C21—H21A	0.9600
C8—H8	0.9300	C21—H21B	0.9600
C9—C10	1.390 (3)	C21—H21C	0.9600
C9—H9	0.9300	C22—N3	1.474 (3)
C10—C11	1.379 (3)	C22—H22A	0.9600
C10—C13	1.511 (2)	C22—H22B	0.9600
C11—C12	1.386 (3)	C22—H22C	0.9600
C11—H11	0.9300	C23—O5	1.206 (2)
C12—H12	0.9300	C23—C24	1.475 (3)
C13—N1	1.451 (2)	C24—C25	1.385 (3)
C13—C14	1.594 (3)	C24—C27	1.386 (3)
C13—H13	0.9800	C25—C30	1.385 (3)
C14—C20	1.501 (3)	C25—C26	1.478 (3)
C14—C18	1.508 (3)	C26—O4	1.209 (2)
C14—C15	1.529 (3)	C27—C28	1.369 (3)
C15—C16	1.544 (3)	C27—H27	0.9300
C15—H15A	0.9700	C28—C29	1.376 (4)
C15—H15B	0.9700	C28—H28	0.9300
C16—N1	1.453 (2)	C29—C30	1.380 (3)
C16—C26	1.528 (3)	C29—H29	0.9300
C16—C23	1.540 (3)	C30—H30	0.9300
C17—N1	1.464 (3)		
			
C6—C1—C2	120.8 (3)	N1—C17—H17C	109.5
C6—C1—H1	119.6	H17A—C17—H17C	109.5
C2—C1—H1	119.6	H17B—C17—H17C	109.5
C3—C2—C1	120.4 (3)	O3—C18—N2	120.46 (18)
C3—C2—H2	119.8	O3—C18—N2	120.46 (18)
C1—C2—H2	119.8	O3—C18—N2	120.46 (18)
C4—C3—C2	119.7 (3)	O3—C18—C14	122.99 (18)
C4—C3—H3	120.2	O3—C18—C14	122.99 (18)
C2—C3—H3	120.2	O3—C18—C14	122.99 (18)
C3—C4—C5	120.4 (3)	N2—C18—C14	116.47 (16)
C3—C4—H4	119.8	O2—C19—N2	121.8 (2)
C5—C4—H4	119.8	O2—C19—N3	120.8 (2)
C6—C5—C4	121.1 (2)	N2—C19—N3	117.35 (16)
C6—C5—H5	119.5	O1—C20—N3	120.9 (2)
C4—C5—H5	119.5	O1—C20—N3	120.9 (2)
C1—C6—C5	117.6 (2)	O1—C20—C14	122.3 (2)
C1—C6—C7	121.4 (2)	O1—C20—C14	122.3 (2)
C5—C6—C7	121.1 (2)	N3—C20—C14	116.58 (16)
C8—C7—C12	117.82 (18)	N2—C21—H21A	109.5
C8—C7—C6	120.4 (2)	N2—C21—H21B	109.5
C12—C7—C6	121.76 (18)	H21A—C21—H21B	109.5
C9—C8—C7	121.2 (2)	N2—C21—H21C	109.5
C9—C8—H8	119.4	H21A—C21—H21C	109.5
C7—C8—H8	119.4	H21B—C21—H21C	109.5
C8—C9—C10	120.63 (18)	N3—C22—H22A	109.5
C8—C9—H9	119.7	N3—C22—H22B	109.5
C10—C9—H9	119.7	H22A—C22—H22B	109.5
C11—C10—C9	118.38 (17)	N3—C22—H22C	109.5
C11—C10—C13	120.06 (18)	H22A—C22—H22C	109.5
C9—C10—C13	121.42 (16)	H22B—C22—H22C	109.5
C10—C11—C12	120.56 (19)	O5—C23—C24	126.94 (19)
C10—C11—H11	119.7	O5—C23—C16	125.32 (18)
C12—C11—H11	119.7	C24—C23—C16	107.73 (16)
C7—C12—C11	121.34 (18)	C25—C24—C27	121.33 (19)
C7—C12—H12	119.3	C25—C24—C23	109.44 (17)
C11—C12—H12	119.3	C27—C24—C23	129.2 (2)
N1—C13—C10	114.30 (16)	C24—C25—C30	120.31 (19)
N1—C13—C14	102.25 (13)	C24—C25—C26	110.15 (17)
C10—C13—C14	114.83 (14)	C30—C25—C26	129.5 (2)
N1—C13—H13	108.4	O4—C26—C25	126.06 (19)
C10—C13—H13	108.4	O4—C26—C16	126.25 (18)
C14—C13—H13	108.4	C25—C26—C16	107.70 (17)
C20—C14—C18	112.55 (15)	C28—C27—C24	118.1 (2)
C20—C14—C15	113.10 (17)	C28—C27—H27	121.0
C18—C14—C15	110.57 (17)	C24—C27—H27	121.0
C20—C14—C13	106.53 (15)	C27—C28—C29	120.7 (2)
C18—C14—C13	109.90 (14)	C27—C28—H28	119.6
C15—C14—C13	103.70 (14)	C29—C28—H28	119.6
C14—C15—C16	106.35 (15)	C28—C29—C30	121.9 (2)
C14—C15—H15A	110.5	C28—C29—H29	119.1
C16—C15—H15A	110.5	C30—C29—H29	119.1
C14—C15—H15B	110.5	C29—C30—C25	117.6 (2)
C16—C15—H15B	110.5	C29—C30—H30	121.2
H15A—C15—H15B	108.6	C25—C30—H30	121.2
N1—C16—C26	113.59 (16)	C13—N1—C16	107.79 (15)
N1—C16—C23	117.38 (16)	C13—N1—C17	114.45 (15)
C26—C16—C23	102.07 (15)	C16—N1—C17	115.16 (15)
N1—C16—C15	103.94 (14)	C19—N2—C18	124.42 (16)
C26—C16—C15	110.39 (16)	C19—N2—C21	117.36 (18)
C23—C16—C15	109.53 (17)	C18—N2—C21	118.21 (18)
N1—C17—H17A	109.5	C20—N3—C19	124.08 (17)
N1—C17—H17B	109.5	C20—N3—C22	118.9 (2)
H17A—C17—H17B	109.5	C19—N3—C22	117.0 (2)
			
C6—C1—C2—C3	−1.4 (6)	C15—C16—C23—O5	−81.1 (3)
C1—C2—C3—C4	0.2 (6)	N1—C16—C23—C24	−141.44 (17)
C2—C3—C4—C5	0.9 (5)	C26—C16—C23—C24	−16.6 (2)
C3—C4—C5—C6	−0.9 (5)	C15—C16—C23—C24	100.42 (18)
C2—C1—C6—C5	1.4 (5)	O5—C23—C24—C25	−166.7 (2)
C2—C1—C6—C7	−179.0 (3)	C16—C23—C24—C25	11.8 (2)
C4—C5—C6—C1	−0.3 (4)	O5—C23—C24—C27	11.6 (4)
C4—C5—C6—C7	−179.9 (2)	C16—C23—C24—C27	−170.0 (2)
C1—C6—C7—C8	135.1 (3)	C27—C24—C25—C30	−0.4 (3)
C5—C6—C7—C8	−45.3 (3)	C23—C24—C25—C30	177.99 (19)
C1—C6—C7—C12	−45.2 (3)	C27—C24—C25—C26	−179.8 (2)
C5—C6—C7—C12	134.4 (2)	C23—C24—C25—C26	−1.4 (2)
C12—C7—C8—C9	2.4 (3)	C24—C25—C26—O4	170.6 (2)
C6—C7—C8—C9	−177.90 (19)	C30—C25—C26—O4	−8.7 (4)
C7—C8—C9—C10	−1.4 (3)	C24—C25—C26—C16	−9.7 (2)
C8—C9—C10—C11	−1.2 (3)	C30—C25—C26—C16	171.1 (2)
C8—C9—C10—C13	174.45 (18)	N1—C16—C26—O4	−37.2 (3)
C9—C10—C11—C12	2.8 (3)	C23—C16—C26—O4	−164.5 (2)
C13—C10—C11—C12	−172.88 (18)	C15—C16—C26—O4	79.1 (3)
C8—C7—C12—C11	−0.7 (3)	N1—C16—C26—C25	143.08 (16)
C6—C7—C12—C11	179.55 (19)	C23—C16—C26—C25	15.7 (2)
C10—C11—C12—C7	−1.9 (3)	C15—C16—C26—C25	−100.64 (19)
C11—C10—C13—N1	−150.00 (17)	C25—C24—C27—C28	0.6 (3)
C9—C10—C13—N1	34.4 (2)	C23—C24—C27—C28	−177.5 (2)
C11—C10—C13—C14	92.3 (2)	C24—C27—C28—C29	0.2 (4)
C9—C10—C13—C14	−83.3 (2)	C27—C28—C29—C30	−1.1 (4)
N1—C13—C14—C20	146.11 (15)	C28—C29—C30—C25	1.2 (4)
C10—C13—C14—C20	−89.53 (19)	C24—C25—C30—C29	−0.4 (3)
N1—C13—C14—C18	−91.69 (16)	C26—C25—C30—C29	178.8 (2)
C10—C13—C14—C18	32.7 (2)	C10—C13—N1—C16	−165.27 (15)
N1—C13—C14—C15	26.53 (19)	C14—C13—N1—C16	−40.56 (18)
C10—C13—C14—C15	150.89 (18)	C10—C13—N1—C17	65.2 (2)
C20—C14—C15—C16	−119.70 (19)	C14—C13—N1—C17	−170.09 (17)
C18—C14—C15—C16	113.04 (19)	C26—C16—N1—C13	158.00 (15)
C13—C14—C15—C16	−4.7 (2)	C23—C16—N1—C13	−83.10 (19)
C14—C15—C16—N1	−18.8 (2)	C15—C16—N1—C13	38.0 (2)
C14—C15—C16—C26	−140.95 (18)	C26—C16—N1—C17	−72.9 (2)
C14—C15—C16—C23	107.42 (19)	C23—C16—N1—C17	46.0 (2)
C20—C14—C18—O3	−153.04 (19)	C15—C16—N1—C17	167.13 (19)
C15—C14—C18—O3	−25.5 (3)	O2—C19—N2—C18	175.49 (19)
C13—C14—C18—O3	88.4 (2)	N3—C19—N2—C18	−7.5 (3)
C20—C14—C18—O3	−153.04 (19)	O2—C19—N2—C21	−3.4 (3)
C15—C14—C18—O3	−25.5 (3)	N3—C19—N2—C21	173.70 (18)
C13—C14—C18—O3	88.4 (2)	O3—C18—N2—C19	171.66 (18)
C20—C14—C18—O3	−153.04 (19)	O3—C18—N2—C19	171.66 (18)
C15—C14—C18—O3	−25.5 (3)	O3—C18—N2—C19	171.66 (18)
C13—C14—C18—O3	88.4 (2)	C14—C18—N2—C19	−11.5 (3)
C20—C14—C18—N2	30.2 (2)	O3—C18—N2—C21	−9.5 (3)
C15—C14—C18—N2	157.77 (16)	O3—C18—N2—C21	−9.5 (3)
C13—C14—C18—N2	−88.34 (19)	O3—C18—N2—C21	−9.5 (3)
C18—C14—C20—O1	153.4 (2)	C14—C18—N2—C21	167.33 (18)
C15—C14—C20—O1	27.2 (3)	O1—C20—N3—C19	−170.2 (2)
C13—C14—C20—O1	−86.1 (2)	O1—C20—N3—C19	−170.2 (2)
C18—C14—C20—O1	153.4 (2)	C14—C20—N3—C19	15.0 (3)
C15—C14—C20—O1	27.2 (3)	O1—C20—N3—C22	8.2 (3)
C13—C14—C20—O1	−86.1 (2)	O1—C20—N3—C22	8.2 (3)
C18—C14—C20—N3	−31.9 (2)	C14—C20—N3—C22	−166.6 (2)
C15—C14—C20—N3	−158.10 (18)	O2—C19—N3—C20	−177.30 (19)
C13—C14—C20—N3	88.62 (19)	N2—C19—N3—C20	5.6 (3)
N1—C16—C23—O5	37.1 (3)	O2—C19—N3—C22	4.3 (3)
C26—C16—C23—O5	161.9 (2)	N2—C19—N3—C22	−172.8 (2)

### Hydrogen-bond geometry (Å, º)

**Table:**

*D*—H···*A*	*D*—H	H···*A*	*D*···*A*	*D*—H···*A*
C29—H29···O2^i^	0.93	2.44	3.269 (3)	149

Symmetry code: (i) *x*+1, *y*, *z*+1.
